# Perceptuo-motor planning during functional reaching after stroke

**DOI:** 10.1007/s00221-017-5058-5

**Published:** 2017-08-12

**Authors:** Margit Alt Murphy, Melanie C. Baniña, Mindy F. Levin

**Affiliations:** 1 Institute of Neuroscience and Physiology, Rehabilitation Medicine, Sahlgrenska Academy, University of Gothenburg, Per Dubbsgatan 14, Plan 3, 41345 Gothenburg, Sweden; 2 School of Physical and Occupational Therapy, McGill University, Montreal, QC Canada; 3 Center for Interdisciplinary Research in Rehabilitation (CRIR), McGill University, Montreal, QC Canada

**Keywords:** End-state comfort effect, Arm, Kinematics, Stroke, Anticipatory motor planning, Visual perception

## Abstract

In healthy young adults, reaching movements are planned such that the initial grasp position on the object is modulated based on the final task goal. This perceptuo-motor coupling has been described as the end-state comfort effect. This study aimed to determine the extent to which visuo-perceptual and motor deficits, but not neglect, due to stroke impact end-state comfort measured as the grasp-height effect. Thirty-four older adults (17 controls, 17 chronic stroke) performed a functional goal-directed two-sequence task with each arm, consisting of reaching and moving a cylindrical object (drain plunger) from an initial to four target platform heights, standardized to body height, in a block randomized sequence. Arm motor impairment (Fugl-Meyer Assessment) and visual–perceptual deficits (Motor-Free Visual Perception Test) were assessed in stroke subjects, and arm and trunk kinematics were assessed in all subjects. The primary outcome measure of the grasp-height effect was the relationship between the grasp heights used at the home position and the final target platform heights. Mixed model analysis was used for data analysis. The grasp-height effect was present in all participants, but decreased in stroke subjects with visuo-perceptual impairments compared to controls. In stroke subjects with sensorimotor impairments alone, indicated by altered kinematics, the grasp-height effect was comparable to controls. This first study examining the grasp-height effect in individuals with stroke provides new knowledge of the impact of visuo-perceptual deficits on movement planning and execution, which may assist clinicians in selecting more effective treatment strategies to improve perceptuo-motor skills and enhance motor recovery.

## Introduction

Movements of the upper limb during daily activities are complex and require the coordination between multiple muscles, joints and body segments in close interaction with environmental and personal constraints (Gibson 2015; Greeno 1994; Shumway-Cook and Woollacott 2012). Visual input, perception and cognitive processing of available information in the environment are involved in the formation of an action plan appropriate for the task (Marteniuk et al. 1987). Several motor control models based on dynamical systems and ecological theory have been proposed to explain different aspects of movement planning and execution of multijoint functional tasks. These models emphasize the importance of the interaction between the individual, environment and task, and explain movements through optimization principles, such as minimizing the degrees of freedom, avoiding biomechanical discomfort or prioritizing comfortable final arm or hand postures (Gielen 2009; Jeannerod et al. 1998; Latash 2012; Rosenbaum et al. 2006; Solnik et al. 2013; Turvey and Fonseca 2009).

Contextual constraints, such as object properties, object location with respect to the body and the goal of the task, impact motor planning and execution (Jeannerod et al. 1998; Marteniuk et al. 1987; Turvey and Fonseca 2009). For example, the kinematics of a pointing task differ from reaching and grasping tasks, in the same way as a reaching movement differs depending on whether a simulated or real object is used (Armbruster and Spijkers 2006; Grafton et al. 1996; Marteniuk et al. 1987). Arm, hand and fingers during reaching are positioned in a way that is sensitive to the location, size, and orientation of the object, but also to how the object will be used, e.g., object affordance (Cohen and Rosenbaum 2004; Jeannerod et al. 1998; Rosenbaum et al. 2009).

One way to study movement planning and perceptuo-motor coupling is by using tasks in which the end-state comfort is manipulated (Rosenbaum et al. 1990). The end-state comfort effect is the tendency of the sensorimotor system to prioritize a comfortable hand and arm position at the end of an object manipulation task rather than at the beginning (Rosenbaum et al. 2012). For example, when young adults were asked to grasp and move an object with a vertical shaft (drain plunger) from its initial position on one platform to a target platform of a different height, they grasped the shaft lower when the plunger was to be moved to higher shelves and higher to move the plunger to lower shelves (Cohen and Rosenbaum 2004). The preferred grasp height was linearly related to the target platform height onto which the object was to be moved, called the grasp-height effect. The authors concluded that young healthy adults planned movements beyond the first phase of the task and that end-state comfort was usually prioritized over initial-state comfort (Cohen and Rosenbaum 2004). Although other tasks requiring positioning of the hand throughout a 180 degree continuous task space may be more sensitive to study perceptuo-motor coupling (Wunsch et al. 2013), these tasks may also require a higher level of motor ability. Thus, in this first study of individuals with stroke, we chose to study the end-state comfort effect of grasp height. The end-state comfort effect is reliably present only beyond the age of 9–10 years (Stockel et al. 2012; Wunsch et al. 2013) and declines with increasing age, which may be associated with reduced cognitive capabilities (Wunsch et al. 2015). Studies investigating the end-state comfort effect in adult clinical populations are few (Rosenbaum et al. 2012; Tan et al. 2012) and how perceptuo-motor coupling may be affected by stroke has not been addressed.

After stroke, several body functions influencing daily activities and consequently participation in social life may be impaired (Beaudoin et al. 2013; Hochstenbach et al. 1998). In addition to commonly reported sensorimotor and cognitive impairments, visual perception is impaired in approximately 30–50% of persons (Beaudoin et al. 2013; Nys et al. 2007). Visuo-perceptual deficits have also been linked to restrictions in activities and participation in daily life (Beaudoin et al. 2013; Titus et al. 1991). Visuo-perceptual deficits differ from visuospatial neglect that is a disorder of attention and awareness primarily resulting from right-sided brain lesions (Proto et al. 2009). Visuospatial neglect influences motor planning and subsequent movement execution (Peters et al. 2015) and may therefore mask more subtle and often non-diagnosed visuo-perceptual deficits (Hermsdorfer et al. 1999).

The design of the study was based on the ecological theory originating from psychology emphasizing that perception of object affordances together with the goal of the task impact motor planning and execution (Gibson 2015; Randerath and Frey 2015; Rosenbaum et al. 2012). Since a large proportion of patients may have non-neglect perceptual deficits after stroke (Proto et al. 2009) that may influence motor learning capacity, valuable information can be obtained by investigating how these deficits affect anticipatory motor planning and movement execution. This knowledge may guide clinicians in the selection of interventions to improve perceptuo-motor skills related to reaching and grasping and enhance upper limb recovery. Thus, the aim of this study was to determine to what extent non-neglect visuo-perceptual deficits and motor deficits in persons with stroke impact prospective motor planning determined by the grasp-height effect in reaches to different target heights.

Our first hypothesis was that the grasp-height effect would be decreased in persons with stroke who have visuo-perceptual deficits, compared to healthy controls and to individuals with stroke without visuo-perceptual impairments. Since perceptual deficits would impact both arms, the effect of these deficits would be most clearly observable when the task is performed with the non-paretic arm as movements of this arm would not be confounded by concurrent motor deficits. Our second hypothesis was that the grasp-height effect would be decreased in persons with stroke who have motor deficits in the paretic arm compared to healthy controls. Motor deficits may limit the choice of grasp location because of limitations in the ability to reach, orient the hand for grasping and to use different types of grasps (Roby-Brami et al. 2003). We anticipated that the effect of motor impairment alone would be most evident in paretic arm of persons without visuo-perceptual deficits. Preliminary results have appeared in abstract form (Alt Murphy and Levin 2016).

## Methods

### Subjects

Seventeen persons with chronic stroke and 17 healthy controls (8 male, mean age 63.7 ± 12.0 years, range 40–78 years) participated. Inclusion criteria for participants with stroke were: presence of unilateral stroke at least 6 months earlier defined according to World Health Organization criteria by imaging or clinical assessment; age between 40 and 80 years; presence of impaired upper limb function (Chedoke McMaster Stroke Assessment Arm and Hand sections ≥4/7); ability to grasp and lift a cylindrical object with the affected arm above shoulder height without pain while standing independently. Participants were excluded if they had unilateral neglect detected by the line bisection test (Menon and Korner-Bitensky 2004) and/or Apples test (Bickerton et al. 2011) or showed signs of apraxia during clinical assessments. Participants were also excluded if they were unable to follow instructions in English and/or French; had other neurological or musculoskeletal conditions affecting upper limb reaching and grasping; or had uncorrected visual acuity problems. Healthy controls were included if they were between 40 and 80 years old. They were excluded according to the same criteria as for individuals with stroke and if they scored <31 out of 36 points on the Motor-Free Visual Perception Test, MVPT (Colarusso and Hammill 2003). Participants were recruited through four local rehabilitation hospital discharge lists and tested at the Research Center of the Jewish Rehabilitation Hospital. All participants provided written informed consent and the study protocol was approved by the Centre for Interdisciplinary Research in Rehabilitation of Greater Montreal (CRIR).

### Clinical evaluation

Upper limb sensorimotor impairment in participants with stroke was assessed using the Fugl-Meyer Assessment for Upper Extremity (FMA-UE, motor 0–66; sensation 0–12) (Duncan et al. 1983; Fugl-Meyer et al. 1975). Spasticity was assessed with the Composite Spasticity Index (0–16) that measures both phasic and tonic stretch reflex activity of the elbow and wrist on which a score of 4 indicates a normal response (Levin and Hui-Chan 1993). A score <26 out of 30 points on the Montreal Cognitive Assessment (MoCA) indicated cognitive impairment (Nasreddine et al. 2005).

Visuo-perceptual deficits were evaluated with the original version of the MVPT, which measures general visual perception independently of motor ability and discriminates between individuals with and without visual perceptual deficits (Cate and Richards 2000; Mazer et al. 1998). The MVPT assesses visual domains of discrimination, figure–ground discrimination, memory, completion of fragmented pictures and spatial relations and has a maximal score of 36. Participants with stroke were stratified according to the presence (≤30 points, *n* = 7) or absence (≥31 points, *n* = 10) of a visuo-perceptual impairment (Mazer et al. 1998). All clinical assessments were performed by an experienced physiotherapist.

### Research setup and procedure

**Fig. 1:**
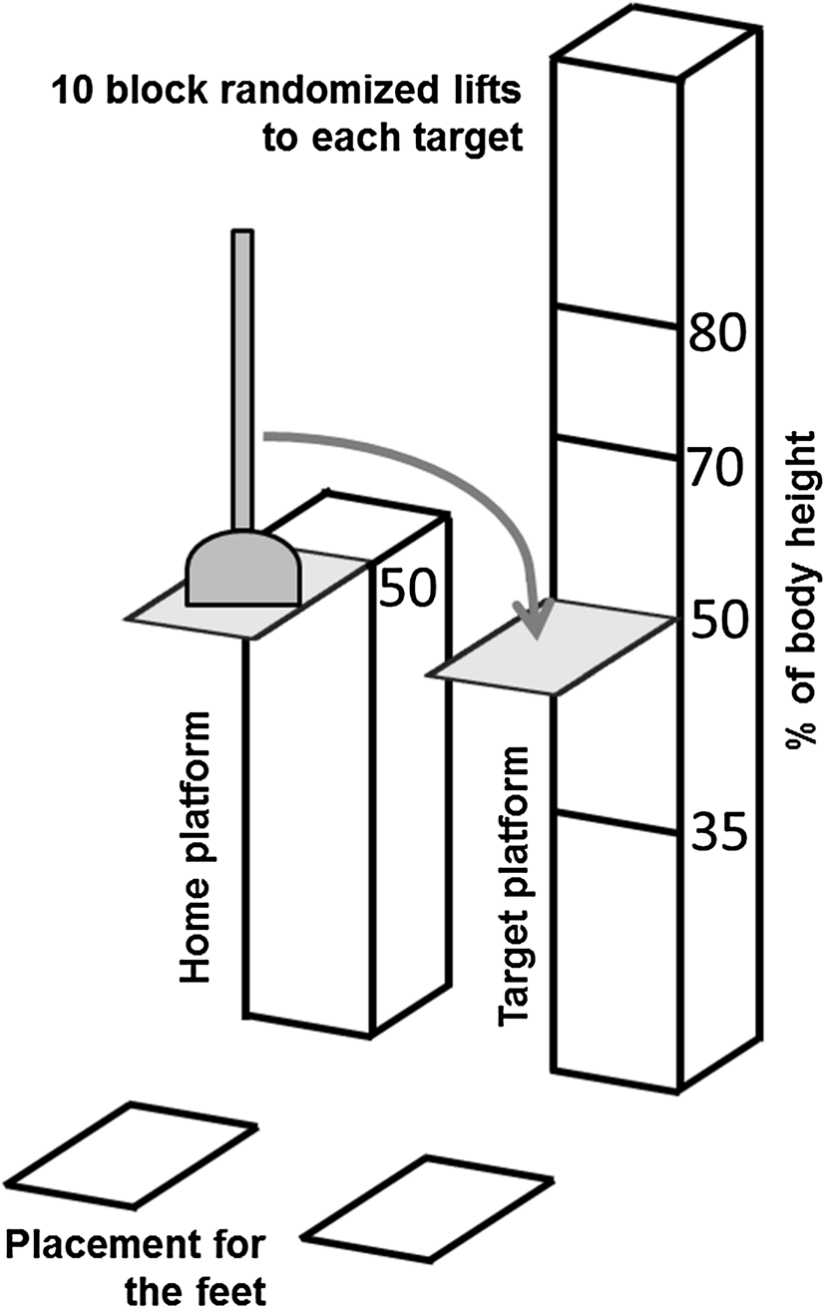
The task incorporated reaching, grasping and moving an object with a vertical wooden shaft (drain plunger) from a “home platform” to four target platforms of different heights (% of body height). The task was repeated in blocks of ten trials per target with the order of target height and starting side randomized across subjects

We used a perceptual motor task similar to that described by Cohen and Rosenbaum (2004) that incorporated reaching, grasping and moving an object with a vertical wooden shaft (drain plunger, 300 g, 42 cm long 2 cm diameter) from a “home” location in front of the contralateral arm of the participant to four target platforms (23 × 23 cm) of different heights in the ipsilateral arm workspace (Fig. 1). The rigid rubber plunger base was 16 cm wide and 7 cm high. The reach-to-move task was tested on both arms.

To be able to compare results from people of different heights and to ensure that reaching extent was similar in all subjects, the locations of the home and target platforms were adjusted to body height, arm length and shoulder width of each participant. The fixed home platform was anterior to the contralateral shoulder at 50% of body height at a distance of 75% arm length. Arm length was defined as the distance between the mid-acromion to the tip of the third finger with the shoulder at 90° and the elbow, wrist and the fingers extended. Target platforms were located laterally to the home platform, anterior to the ipsilateral shoulder, at four different heights set at 35, 50, 70 and 80% of body height.

In the initial position, participants stood with their arm alongside their body. After a verbal “go” command, they reached to grasp the plunger and move it from the home platform to a selected target platform at a comfortable self-selected speed. Participants were asked to complete the task as naturally as possible using a whole hand (palmer) grasp. No other instructions were given. To perform the task, the plunger had to be grasped at some point on the vertical shaft above the rubber base. The highest target was placed so that the subject had to lift the plunger by extending their arm above shoulder level. The lowest target was placed at a level where it was possible to move the plunger without flexing hips or knees. Thus, targets were placed within the full range of shoulder positions. After each trial, the plunger was brought back to the home platform by the examiner, who grasped the base of the plunger with both hands to avoid any observational learning effect. The procedure was repeated in blocks of 10 trials per target with the order of target height and starting side randomized across subjects. Prior to each block, participants performed one practice trial before data capture to ensure that they had understood the instructions and were able to reach the target platform. In total, 40 trials (4 × 10) were recorded for each arm and the entire testing session took approximately 90 min including rest periods.

### Kinematic analysis

A 3D optical motion tracking system was used to sample data at 120 Hz (6-camera Optotrak Certus Motion Capture System, Northern Digital Corp., Waterloo, ON, Canada). Ten active markers (infrared emitting diodes) were placed on the tested hand (third metacarpal head), wrist (ulnar styloid process), elbow (lateral epicondyle), right and left shoulder (anterior–superior acromion), thorax (mid-sternum), and right and left ASIS. Two additional markers were placed on the base of the plunger. In addition, a rigid body with three markers was placed on the upper 1/3rd of the sternum to track 3D trunk movement. NDI First Principles™ software was used for data collection and management.

Kinematic data were analyzed using custom-made Matlab software (Matlab R2013b, The Mathworks Inc, Natick, CA, USA) and filtered using a 10-Hz low-pass Wiener filter which accurately preserves the timing and shape of the signal (Wiener 1970). Since the task goal influences the movement kinematics (Marteniuk et al. 1987), we asked subjects to grasp the plunger at the home position and move it to the target platform as a continuous gesture. However, only the first reach-to-grasp movement, from the initial position to the time of grasping of the plunger, was analyzed. Movement beginning and end were defined as the times at which the tangential velocity of the hand marker exceeded or was less than 5% of the maximal velocity for at least 50 ms. Movement time and peak hand tangential velocity were calculated. Arm and trunk kinematics were characterized by shoulder horizontal adduction/abduction, elbow flexion/extension, and axial trunk rotation angles obtained at the end of the reach phase. Shoulder horizontal adduction was measured as a positive angle of the vector between the acromion and elbow markers projected horizontally and defined as 0° when the arm was abducted laterally in line with the shoulders. Elbow angle was determined by the angle between the vectors joining the shoulder/elbow and elbow/wrist markers with full extension defined as 0°.

To perform the task correctly, the individual had to choose the height of the initial hand placement on the plunger so that the hand position when the plunger was moved to the final target height would be both effective and comfortable. Thus, the presence of a grasp-height effect was defined as a linear relationship between the grasp heights used at the home position and the final target platform heights. A higher grasp at the home position was expected for moves to lower target heights, and a grasp closer to the base of the plunger at the home position was expected for moves to higher target heights. Initial grasp height was calculated as the vertical distance in mm from the marker located on the base of the plunger to the hand marker.

### Statistical analysis

The SPSS (Statistical Package for Social Sciences, version 22) was used for independent *t* tests and Chi-square tests to determine differences in age and sex between stroke and healthy groups, respectively. Differences in clinical characteristics (motor, sensation, spasticity, cognition) in the subgroups of stroke subjects with and without visuo-perceptual impairments were determined using Mann–Whitney *U* tests. Comparisons between kinematics of the right and left arms of healthy controls were done with paired-sample *t* tests or Wilcoxon’s signed rank tests (in case of non-normal distribution). Differences in kinematics between individuals with stroke and healthy controls (left arm) were investigated with one-way analyses of variance (ANOVA) with criterion of significance of *p* < 0.05. Three different analyses were performed comparing three groups at a time: (1) controls and the affected and less-affected arm of individuals with stroke; (2) controls and the stroke subgroups (with and without visuo-perceptual impairment) for movements made with the more-affected arm; (3) controls and the same stroke subgroups for movements made with less-affected arms. Post hoc comparisons using the Bonferroni corrections were used.

A split-plot design was used for the analyses to determine the effect of target platform height on initial grasp height (dependent variable) on the plunger across different groups. Subjects were considered as main plots and target heights as subplots, since the order of target heights was randomized for each subject. The groups and target heights were considered as fixed factors and the subjects as random factors. Interaction terms between all factors were included in the model. The model was adjusted for age, sex and trial order and the estimates and *F* values were compared with an unadjusted model. For the stroke group, the influence of cognitive impairment and lesion side on the effect of target height on grasp height was evaluated within the mixed models analysis. The mixed SAS-procedure (Proc Mixed, SAS Institute Inc., Cary, NC, USA, version 9.3) with restricted maximum likelihood (RELM) estimation method was used. The RELM is recommended for split-plot designs as it yields good results for various relative magnitudes of the variance components even with small and second-order designs (Goos 2006; Kenward and Roger 1997). The assumptions for the analyses were verified by residual plots of predicted values, histograms and q–q plots.

To verify whether the grasp-height effect was decreased in persons with stroke compared to controls, group and interaction effects between group and target height were investigated. Absolute grasp heights for each target height condition as well as the pairwise grasp height differences (within groups) across target platform heights were calculated and compared between controls and subjects with stroke. The grasp height differences in subjects with stroke were compared to controls in a piecewise linear analysis between each pair of target height condition. In addition, grasp height differences (within groups) between each pair of adjacent targets were compared to no (zero) difference. Subsequently, pairwise slopes of grasp heights between the low and high target platform heights were plotted to verify whether the grasp-height effect was decreased in stroke subjects compared to controls. In all comparisons of grasp heights, a Bonferroni *p* value correction was used (*p* < 0.025) to correct for testing the two arms (affected and less-affected) in the whole group analysis; and the two stroke subgroups (with or without perceptual deficits) in the subgroup analyses.

Group effects were evaluated between values from the left arm of controls and the affected and less-affected arm of individuals with stroke. In addition, we evaluated differences between stroke subgroups (stroke subjects with and without visuo-perceptual impairment) for movements made with the more-affected and less-affected arms. To address the first hypothesis, data from the subgroup of stroke subjects with visuo-perceptual impairments performing the task with the less-affected arm (predominant visuo-perceptual impairment) were compared to controls. To address the second hypothesis, data from the subgroup of stroke subjects with no visuo-perceptual impairments using the affected arm (predominant sensorimotor impairment) were compared to controls.

Spearman rank order correlations (rho) were used to identify relationships between the grasp-height effect (measured as grasp height difference between the 35 and 70% targets) and clinical variables of sensation, spasticity and motor impairment for the affected arm and cognition and visuo-perceptual impairment for the less-affected arm. Correlations with visuo-perceptual impairments were only tested for the whole stroke group.

## Results

Clinical characteristics of individuals with stroke are shown in Table 1. All but one participant in each group were right-hand dominant. There were no differences in age (*p* = 0.377) or sex (*p* = 0.078) between participants with stroke and controls. No clear difference in the lesion location was found between individuals with or without visuo-perceptual impairment due to stroke. MoCA scores were significantly lower in the stroke subgroup with visuo-perceptual impairment compared to those without impairment (*p* = 0.007). Since grasp heights and other kinematic measures did not differ between the left and right arms of control subjects, the left arm of controls was used for all comparisons with persons with stroke.

**Table 1:** Demographic and clinical characteristics of participants with stroke

No.	Sex/side	Type	Location	Age (years)	Time since stroke (months)	FMA (0–66)	Sens (0–12)	CSI (0–16)	MVPT (0–36)	MoCA (0–30)
1	M/L	Inf	Subcortical, basal ganglia	58	24	29	11	8	33	25
2	M/R	Hem	Subcortical, basal ganglia	54	65	33	8	7	27	24
3	M/R	Inf	Subcortical	60	31	35	12	6	27	24
4	M/L	Hem	Cortical, subarachnoid	66	240	35	1	9	32	26
5	M/L	Hem	Subcortical, basal ganglia	48	61	36	5	11	34	28
6	M/L	Hem	Cortical, MCA, subarachnoid	51	62	43	2	11	28	19
7	F/L	Inf	Cortical/sub-cortical, parietal/insular	71	23	52	3	5	31	24
8	M/R	Inf	NA	57	24	53	10	10	30	25
9	F/L	Inf	Subcortical, basal ganglia	52	84	54	12	8	29	23
10	F/R	Inf	NA	68	85	54	10	9	34	29
11	M/R	Inf	Subcortical, head of caudatus, internal capsule	70	61	58	12	5	35	26
12	M/R	Inf	Subcortical, thalamus, pons	48	11	58	10	6	36	28
13	M/R	Inf	Subcortical, MCA, sylvian	74	38	59	4	4	27	21
14	M/L	Inf	Subcortical, pons	56	13	59	10	5	33	26
15	M/R	Inf	Subcortical, external capsule	68	24	61	9	7	23	22
16	M/R	Inf	Cortical/sub-cortical, MCA, sylvian	57	11	63	12	5	34	25
17	F/L	Hem	NA	57	66	65	12	5	34	24
Mean				59.7	54.3	49.8	8.4	7.1	31.0	24.6
SD				8.3	54.0	11.9	3.9	2.3	3.6	2.5
Stoke subgroup with VPI, MVPT ≤30, *n* = 7										
Mean				59.4	46.9	48.3	8.1	7.6	27.3	22.6*
SD				8.6	23.5	11.3	3.8	2.4	2.2	2.1
Stroke subgroup with no VPI, MVPT ≥31, *n* = 10										
Mean				59.9	59.5	50.9	8.6	6.8	33.6	26.1*
SD				8.5	68.9	12.8	4.1	2.3	1.4	1.7

*Side* stroke lesion side, *M* male, *F* female, *R* right, *L* left, *Inf* infarct, *Hem* hemorrhage, *NA* not available, *MCA* middle cerebral artery, *FMA* Fugl-Meyer Assessment for Upper Extremity, *Sens* sensation, *MVPT* Motor-Free Visual perception Test, *CSI* Composite Spasticity Index, *MoCA* Montreal Cognitive Assessment, *SD* standard deviation, *VPI* visuo-perceptual impairment

* Significant difference between the stroke subgroups (*p* = 0.007)

### Reach-to-grasp kinematics in stroke and healthy controls

**Fig. 2:**
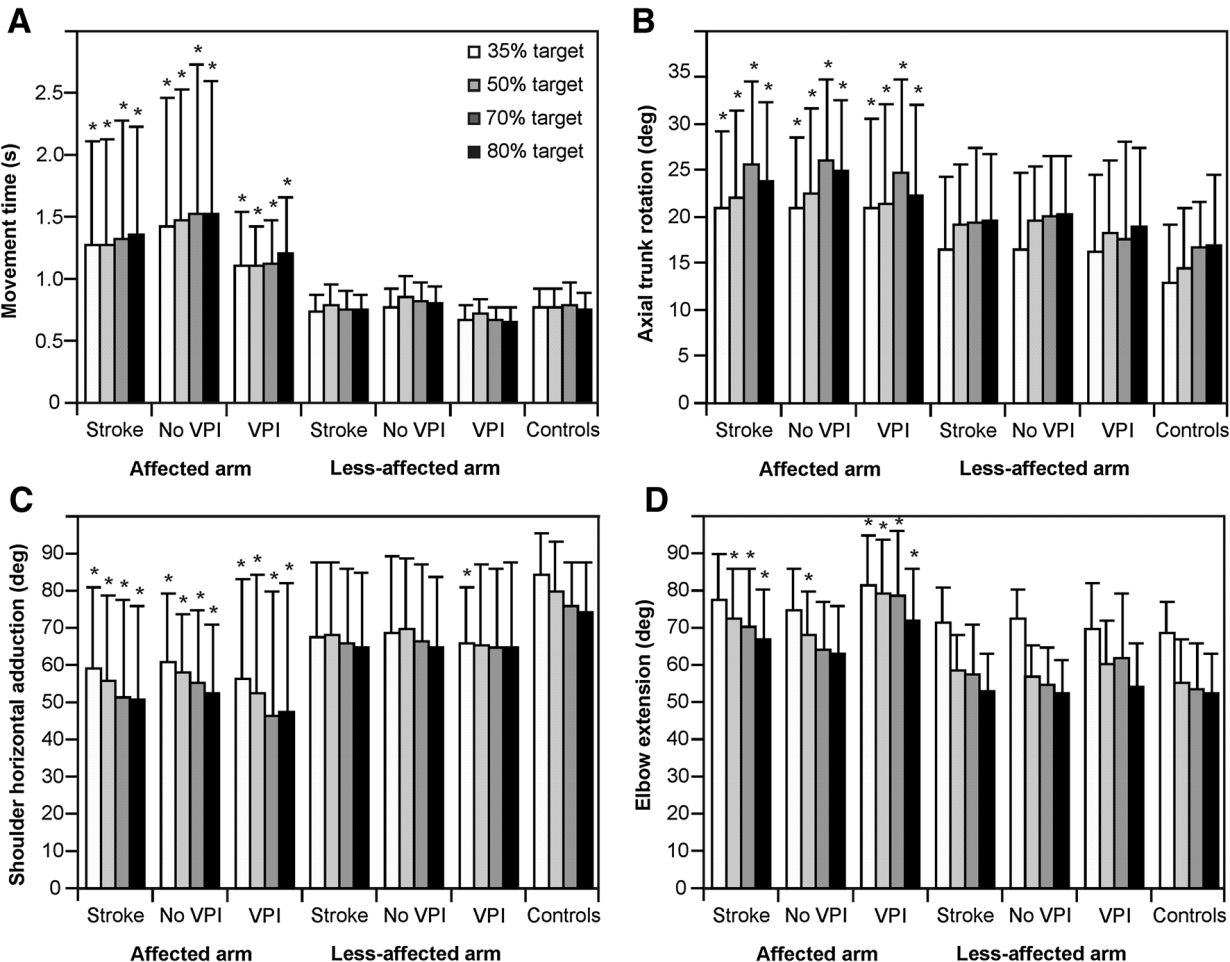
Histograms of mean (SD) kinematic variables of the reach-to-move task for the stroke group, the stroke subgroup with no visuo-perceptual impairments (no VPI), the stroke subgroup with visuo-perceptual impairments (VPI) and the left arm of the healthy control subjects. Movement time of reaching, arm and trunk angle position data at the end of the reach phase are shown. Full elbow extension and horizontal arm abduction laterally in line with the shoulders were defined as 0°. *p < 0.05 compared to healthy controls

Movements of the affected arm in individuals with stroke, regardless of the presence of visuo-perceptual impairments, were significantly slower (*F*_2,48_ = 5.6–7.9, *p* < 0.05, Fig. 2a) and had lower peak velocities (affected arm 1335–1597 mm/s, less-affected arm 1789–1996 mm/s, controls 1769–2083 mm/s, *F*_2,48_ = 5.0–7.4, *p* < 0.05) compared to the less-affected arm and left arm of controls. Individuals with stroke also used more axial trunk rotation (*F*_2,48_ = 3.4–4.1, *p* < 0.05), less shoulder horizontal adduction (*F*_2,48_ = 5.7–8.5, *p* < 0.05) and less elbow extension (*F*_2,48_ = 6.5–10.1, *p* < 0.05) of the affected arm at the time of grasping compared to controls (Fig. 2b–d). Kinematics of the less-affected arm of stroke subjects, regardless of the presence of visuo-perceptual impairments, did not differ from those of controls except for shoulder horizontal adduction for the 35% target height in the subgroup with visuo-perceptual impairments (Fig. 2).

### Grasp-height effect in stroke (whole group) and healthy controls

**Fig. 3:**
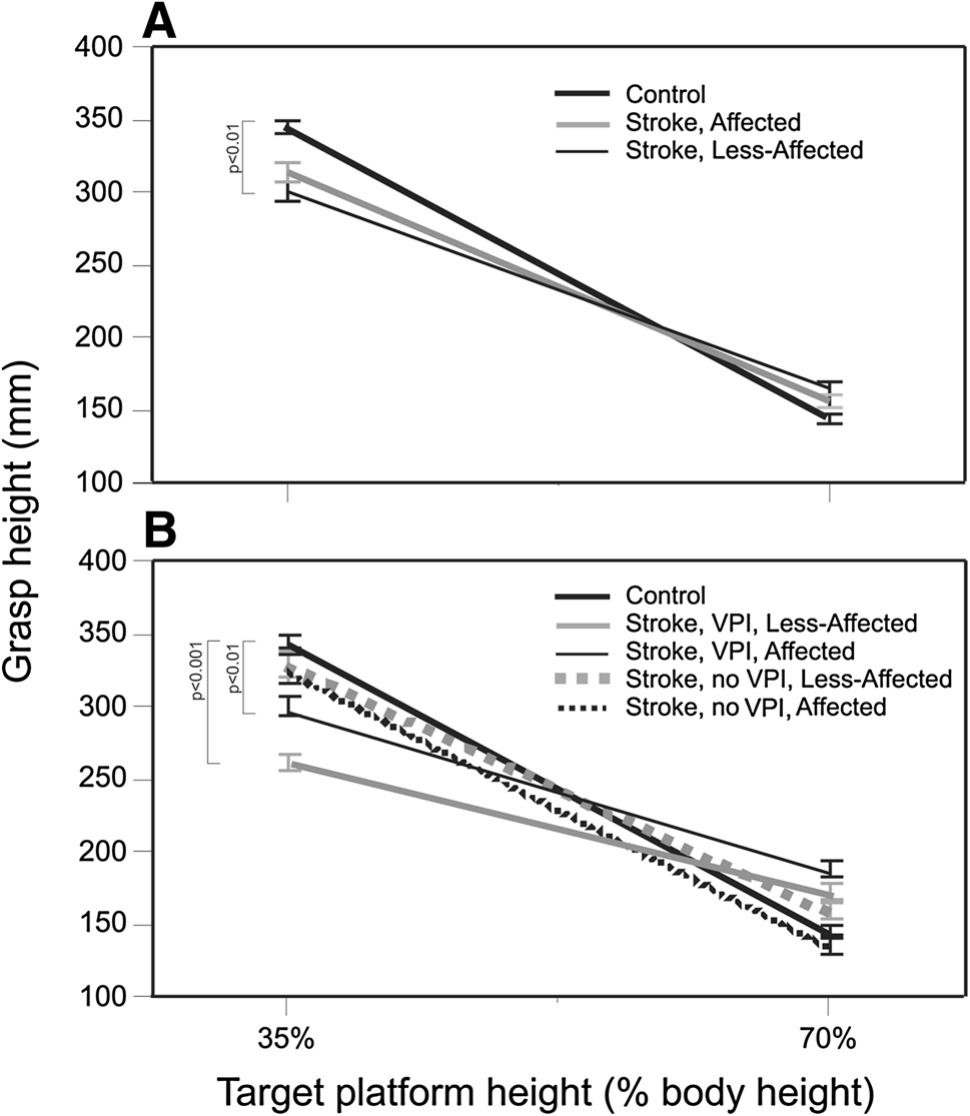
Linear relationships, plotted as slopes, of grasp height values for 35 and 70% target platform heights for **a** the less-affected (*n* = 17) and affected (*n* = 17) arms of subjects with stroke, and for **b** subgroups of stroke subjects with (VPI, *n* = 7) and without visuo-perceptual impairment (no VPI, *n* = 10) together with the left arm of healthy controls (control, *n* = 17). Significant differences between stroke groups/subgroups compared to controls are indicated

The initial grasp height at the home position varied with target platform height both in individuals with stroke (affected arm and non-affected arm) and in controls (*F*_3,48_ = 105, *p* = 0.0001) such that a higher grasp on the plunger shaft was used for moves to lower target heights, and a grasp closer to the base of the plunger was used for moves to higher target heights. Individuals with stroke varied their grasp height according to the grasp-height effect, but to a lesser degree compared to controls. The overall effects are illustrated in Fig. 3a. Since there were no differences in mean grasp heights in any group for the 70 and 80% target platform heights (*p* > 0.025), linear pairwise plots were constructed for grasp height values between the 35 and 70% target heights. The slope of this relationship was lower for the whole stroke group when using their less-affected arm compared to arms of healthy subjects (*p* = 0.009; Fig. 3a; Table 2B). The absolute grasp heights used for moves to each target platform height and the differences in grasp heights between target heights are listed for all groups in Table 2.

**Table 2:** Absolute grasp heights used in movements to each target platform height (A) and differences in grasp heights between target heights conditions (B) in individuals with stroke and healthy controls

Target height (%)	Stroke, less-affected arm (mm)			Stroke, affected arm (mm)			Controls (*n* = 17)
	All (*n* = 17)	VPI (*n* = 7)	No VPI (*n* = 10)	All (*n* = 17)	VPI (*n* = 7)	No VPI (*n* = 10)	
(A) Absolute grasp heights at each target height condition, mean (SD)							
35	300.8* (82.6)	261.5** (51.6)	329.0 (80.3)	314.3 (81.1)	300.0 (60.1)	325.3 (81.9)	344.7 (59.5)
50	202.5 (66.9)	208.2 (52.5)	198.5 (75.6)	231.2* (68.8)	254.0** (40.4)	215.3 (79.4)	180.4 (63.9)
70	164.3 (59.7)	171.2 (48.8)	159.5 (66.1)	155.6 (49.4)	187.7 (44.5)	135.7 (41.2)	144.3 (49.4)
80	154.6 (49.0)	167.3 (41.0)	145.3 (52.5)	152.0 (53.4)	166.9 (45.0)	142.5 (56.3)	123.4 (32.0)

Between targets (%)	Stroke, less-affected arm (mm)			Stroke, affected arm (mm)			Controls (*n* = 17)
	All (*n* = 17)	VPI (*n* = 7)	No VPI (*n* = 10)	All (*n* = 17)	VPI (*n* = 7)	No VPI (*n* = 10)	
(B) Differences in grasp heights between targets, mean (SD)							
35–50	98.3** (80.3)	53.3*** (33.3)	130.5 (93.5)	83.1*** (72.8)	45.9*** (55.7)	110.0* (82.4)	164.4 (67.2)
35–70	136.5** (85.4)	90.3*** (52.2)	169.5 (97.0)	158.7 (83.0)	112.2** (68.2)	189.6 (92.1)	200.4 (67.6)
35–80	146.1** (85.8)	94.2*** (39.6)	183.7 (100.4)	162.4* (84.8)	133.0** (94.2)	182.9 (87.9)	221.3 (56.1)
50–70	38.2 (57.2)	36.9 (41.1)	39.0 (68.6)	75.5 (64.2)	66.3 (18.6)	79.6 (80.1)	36.0 (61.0)
70–80	9.7 (29.4)	3.9 (28.0)	14.2 (31.6)	3.7 (22.3)	20.8 (48.9)	−6.8 (28.7)	20.9 (33.0)

*VPI* visuo-perceptual impairment

* *p* < 0.025, ** *p* < 0.01, *** *p* < 0.001 compared to controls

The mixed models analysis indicated a significant interaction between target platform heights and groups (affected and less-affected arms in stroke, left arms in controls; *F*_6,48_ = 3.93, *p* = 0.003; Fig. 3a). Age (*p* = 0.49), sex (*p* = 0.70), cognitive impairment (*p* = 0.94), lesion side (*p* = 0.80) and trial order (*p* = 0.92) were not significant when added to the mixed analysis model.

### Visuo-perceptual impairments and grasp-height effect

Similar to the whole group analysis, there was a significant interaction between target height and group for the four stroke subgroups and the left arms in controls (Fig. 3b; *F*_12,45_ = 2.86, *p* = 0.005). Slopes of grasp heights between the 35 and 70% target platform heights were lower for the subgroup of stroke subjects with visuo-perceptual impairments compared to controls when the task was either performed with the less-affected (*p* = 0.0009) or the affected arm (*p* = 0.005; Fig. 3b; Table 2B). Thus, in line with the first hypothesis, the grasp-height effect was decreased in persons with stroke who had visuo-perceptual deficits compared to healthy controls. This effect was observable in both arms, although the slope was decreased most when the task was performed with the less-affected arm.

### Motor impairments and grasp-height effect

According to the second hypothesis, the level of motor impairment was expected to influence the grasp height primarily in stroke subjects without visuo-perceptual deficits performing the task with the affected arm. In this subgroup, absolute grasp heights used for each target condition did not differ from controls (Table 2A). The slopes for grasp heights between the 35 and 70% target platform heights were not different from controls when the task was either performed with the less-affected (*p* = 0.21) or affected arm (*p* = 0.65; Table 2B; Fig. 3b). Thus, in individuals without visuo-perceptual impairments, the grasp-height effect was not different from controls irrespective of which arm was used to perform the task.

### Grasp-height effect and other clinical impairments

Sensory, spasticity and motor impairments after stroke were not correlated with the grasp-height effect for affected arms and cognitive impairments were not correlated with the grasp-height effect for less-affected arms in the whole group and for subgroups with or without visuo-perceptual impairments. In the whole stroke group, the visuo-perceptual impairments were not correlated with the grasp-height effect.

## Discussion

This study investigated whether and to what extent visuo-perceptual and motor deficits due to stroke influence motor planning and execution in sequential reaching and moving an object to different heights in the arm workspace. Movement planning of this two-sequence task was reflected in the relationship between the initial grasp height on the plunger shaft and the final position of the target platform to which the plunger was to be moved. In young healthy adults, a linear relationship between the grasp height and the target platform height has been observed (Cohen and Rosenbaum 2004; Rosenbaum et al. 2006). This relationship, described as the grasp-height effect, represents an efficiency constraint in movement planning, in which the initial grasp height is modulated with respect to the final goal of the task (Rosenbaum et al. 2012).

The grasp-height effect was present in all groups and subgroups, but the extent of this effect was decreased in the subgroup of stroke subjects with visuo-perceptual deficits. The decrease in grasp-height effect was present irrespective of the arm used for the task, but it was most evident when the task was performed with the less-affected non-paretic arm, for which the motor impairments did not interfere with movement planning and performance. In the stroke participants with visuo-perceptual deficits, a lower grasp height was used for moving the plunger to the lowest target height and a higher one was used for all other target heights compared to controls (Table 2). In other words, the variation in grasp height for moving the plunger to different target heights was smaller in this group.

Despite the high prevalence of visuo-perceptual deficits in patients with stroke, it is unclear how these deficits might affect motor planning and execution. Studies investigating perceptuo-motor coupling in clinical populations are few and visuo-perceptual deficits are generally not reported. The strength of the present study is the use of an objective and sensitive method (i.e., kinematics) for determining the exact position of the grasp on the object and to control for whether the existing movement impairments influenced the grasp-height effect. Furthermore, testing both the affected and less-affected arms of the stroke subjects allowed us to identify the grasp-height effect separately for those with sensorimotor or visuo-perceptual impairments. Our findings that visuo-perceptual impairments, particularly observable for movements not confounded by motor deficits, influenced the grasp-height effect, add valuable new information about deficits in movement planning and execution in clinical populations. The study also adds new knowledge of movement planning in individuals with more subtle visuo-perceptual impairments, since those with visual neglect were not included.

The end-state comfort effect during a bar rotation task with the less-affected arm was decreased in adults with visual agnosia and cerebral palsy (Craje et al. 2009; Dijkerman et al. 2009). Individuals with stroke and visuo-spatial disorders affecting the perception of line orientations, showed a decreased end-state comfort effect while performing a two-sequence bar rotation task with the ipsilesional arm (Hermsdorfer et al. 1999). However, this decreased effect may have been partly accounted for by the presence of neglect and hemianopia in their study participants. In contrast, in individuals with stroke and apraxia, grip selection was similar to controls, although movement execution was slower and uncoordinated (Hermsdorfer et al. 1999). In our study, persons with neglect were excluded, but a decreased grasp-height effect was still observed in individuals with visuo-perceptual impairments, which implies that more subtle visuo-perceptual deficits may also influence grip selection and the end-state comfort effect.

The end-state comfort effect was also decreased in older adults between 71 and 80 years compared those aged 60–70 years, in whom the end-state comfort effect was comparable to young adults (Wunsch et al. 2015). An increase in task complexity, requiring bimanual manipulation was also shown to decrease the end-state comfort effect in older adults (Wunsch et al. 2015). These authors concluded that the decrease in the older group may be associated with reduced cognitive capabilities. In the current study, the mean age of the control and stroke groups was 64 and 60 years, respectively, and the task was unilateral. Thus, the cognitive demand for the plunger transport task was relatively low and the risk of possible decline related to age was relatively small. In addition, neither age nor cognitive impairment significantly influenced the grasp-height effect in the current study. Increased demand on precision has also been suggested to influence the end-state comfort effect such that a more comfortable hand or grasp position is selected in the phase in which the highest precision is needed (Kunzell et al. 2013; Rosenbaum et al. 2012). However, this was unlikely to have influenced our results since in our task, the requirements for precision were low, as the target platform was larger than the base of the plunger and the plunger itself had a steady base.

Anticipatory movement planning of functional reach-to-grasp movements can be investigated in several ways. In the current study, the grasp-height effect was used to gain insight into motor planning and action selection in accordance with the end-state comfort effect. Tasks requiring a larger degree of object rotation (i.e., 180° rotations) have been suggested to be more sensitive to the end-state comfort effect in healthy populations (Wunsch et al. 2013). On the other hand, when the hand-orientation and grasp-height effect were investigated together in the same task in young adults, end-state comfort was prioritized both for the grasp orientation and height (Cohen and Rosenbaum 2011). Since 180° rotations may be challenging for individuals with stroke due to motor deficits, we chose to study the end-state effect of grasp height in this first study with individuals after stroke. End-state comfort based on orienting a dowel and rotating a card has also been used to evaluate treatment effects on the paretic arm in persons with chronic stroke (Tan et al. 2012). Persons with stroke used a grasp posture consistent with the end-state comfort effect in most trials (68%), but less frequently than controls (91%). Tan et al. (2012) also reported that the number of trials consistent with the end-state comfort effect increased after constraint-induced movement therapy. Whether the diminished end-state comfort effect was a result of impaired motor planning or a combined effect of motor impairment and planning deficits, however, cannot be determined since the task was only performed with the more affected upper limb. Thus, the motor impairment may have obscured possible effects of visuo-perceptual deficits. In our study, visuo-perceptual deficits, and not motor impairments, accounted for deficits in motor planning in our subgroup of stroke subjects. This is supported by findings that the grasp-height effect was decreased in all stroke subjects with visuo-perceptual impairments regardless of which arm was used (paretic or non-paretic) and that the grasp-height effect was preserved in stroke subjects without visuo-perceptual deficits when using the more-affected arm.

A growing body of research suggests hemispheric specialization for different aspects of movement (Haaland et al. 2004; Mani et al. 2013; Sainburg and Schaefer 2004; Schaefer et al. 2007). Hemisphere-specific effects in end-point control in reaching with the paretic arm were found in individuals with stroke after right but not left brain damage (Stewart et al. 2014). These differences could not be explained by age, time post-stroke, motor function or apraxia, but instead, may be related to differences in visual-motor processing, since visual perception deficits, assessed using the MVPT, were larger in the right brain damaged group (Stewart et al. 2014). The right hemisphere has been suggested to play a dominant role in processing visuo-spatial effects of goal-directed movements (Hermsdorfer et al. 1999), whereas each hemisphere may be specialized for different aspects of task execution (Mani et al. 2013; Mutha et al. 2012; Sainburg and Schaefer 2004). Studies in individuals with stroke are, however, few and future studies with selected groups are warranted. In the current study, the side of the lesion did not influence the grasp-height effect when added to the analyses, which partly may be related to the small sample size and the low task difficulty. Larger studies directly investigating hemispheric differences including kinematic analysis and taking visuo-perceptual deficits into account may provide more accurate information regarding hemispheric lateralization.

Results from the subgroup analysis should be interpreted with caution, partly because of the small sample sizes, and partly because other impairments, such as sensory impairments or apraxia not observed during clinical assessments (Randerath et al. 2009), may also have affected the grasp-height effect. The grasp-height effect showed, however, no correlations with sensory impairments, spasticity or cognitive impairment. It is recognized that deficits in different MVPT domains including the ability to discriminate and recall dominant features of an object, distinguish it from the background, orient one’s body in space and perceive the object position in relation to oneself and to other objects, might all have affected movement planning of functional reaching in the current study. However, since individual domains of visual perception of the MVPT have not been validated, the results cannot be related to specific perceptual domains, which is a limitation of the study. Future studies should include a more specific and comprehensive assessment of perceptual and cognitive impairments, preferably together with lesion analysis (Randerath et al. 2010), to help identify the relationships between these impairments and motor behavior more precisely. The results of this study can only be applied to individuals with moderate to mild motor impairment who are in the chronic stage of stroke and independent in standing during functional tasks. In the current study, only linear modeling for grasp height data was used and future studies should consider data collection from a larger number of possible target heights and nonlinear modeling for investigating the grasp-height effect both in healthy and clinical populations.

Visuo-perceptual deficits in individuals with stroke impacted planning and selection of movements of a two-sequence functional task. This suggests that in clinical settings, patients should practice tasks involving planning over two steps that require different hand orientations, in order to facilitate improvement of perceptuo-motor skills. Furthermore, to develop the ability to modulate the grasp according to object affordances, practice of reaching tasks to different locations in the arm workspace within a training session is recommended. An increased understanding of underlying motor control processes would facilitate development and implementation of effective interventions to improve perceptuo-motor skills in individuals with stroke.
